# A biopsychosocial examination of chronic back pain, limitations on usual activities, and treatment in Brazil, 2019

**DOI:** 10.1371/journal.pone.0269627

**Published:** 2022-06-03

**Authors:** Flavia Cristina Drumond Andrade, Xiayu Summer Chen

**Affiliations:** School of Social Work, University of Illinois at Urbana-Champaign, Urbana, Illinois, United States; Emory University, School of Public Health, UNITED STATES

## Abstract

**Background:**

Chronic back pain is prevalent in Brazil, leading to enormous healthcare costs and social burdens. It also disproportionately affects low-income and less-healthy people.

**Objectives:**

This study examines the associations of chronic back pain with biological, psychological, and social factors; how it limits usual activities; and how chronic back pain influences the use of treatment services.

**Methods:**

Using Brazil’s National Health Survey (PNS-2019), multivariate logistic regressions were conducted to examine how biological, psychological, and social factors correlate with chronic back pain, limitations on usual activities, and pain treatment.

**Results:**

PNS-2019 data showed that 23.4% (95% CI 22.8–24.0) of Brazilian adults aged over 20 reported back pain. A higher prevalence of chronic back pain was associated with biological factors (older age, being female, overweight or obese, current smoking, and having more chronic conditions), lower social conditions (low education, low per capita household income, non-married, and living in rural areas), and poor psychological health (more depressive symptoms). Chronic back pain is more likely to limit usual activities among those with low social conditions (lower education, lower income), poor physical and behavioral health (obese, current smokers, and those with a greater number of chronic conditions), and worse psychological health (more depressive symptoms). However, married people and those who do not consume alcohol were also more likely to report limited activities. Among those with back pain, 68% received at least one form of treatment. Those with intense limitations on their usual activities were 2.2 times as likely to report treatment. People with higher social conditions (higher income, college education, and private health insurance) were more likely to receive treatment.

**Conclusion:**

The results show significant biological, psychological, and social disparities in the prevalence of chronic back pain in Brazil. The findings point to the need for tailored policies and prevention programs with attention to vulnerable groups. Even though Brazil has universal health care, those with better socioeconomic conditions are more likely to receive treatment.

## Introduction

Chronic back pain is a major health concern throughout the world, and it has been the leading cause of non-fatal health loss for nearly three decades [1]. It is highly prevalent and has high healthcare and lost productivity costs [2, 3]. The high estimated healthcare and social burden in Brazil are enormous [4]; estimates put it at $2.2 billion over 2012–2016 [2]. In Brazil, the rate has increased from 13.2% in 2003 to 18.5% in 2013 [5, 6]. According to the Global Burden of the Disease, low back and neck pain were the leading causes of years lived with a disability in Brazil between 1990 and 2016 [4]. The age-standardized rate due to low back and neck pain declined by 4.4% in this period, from 11,355 to 11,012 per 100,000 [4].

Previous studies have indicated that biological factors and health behaviors predict chronic back pain. These include increased age [5, 7–9], sex [10–12], history of smoking [5, 13], alcohol abuse [14], physical activity [5, 15], number of chronic diseases [5, 16, 17], and higher body mass index (BMI) [5, 18]. Social conditions, such as lower education level [8, 19–21], occupation [8, 19, 22], lower income[8, 9, 23], and residential area [5, 17, 24] also show associations, as do psychological factors like depression and depressive symptoms [17, 18, 25]. People living in lower socioeconomic status and rural areas are often less likely to access multidisciplinary pain management for chronic back pain than their counterparts [26]. Studies in Brazil have shown associations between chronic back and biological and behavior factors such as older age, being female, and smoking history [5, 12]; and lower social conditions, like having a lower education level [27] and living in a rural area [5]. A study of treatment in Brazil showed that 43.9% of older participants did not receive any treatment despite the high prevalence of chronic back pain, that those who did receive treatment typically used medication and/or physical therapy/exercise, and that education, socioeconomics, and urban residence increased access to physical therapy/exercise while medication was more prevalent among less educated, poorer, and rural older adults [28]. But these studies are limited in that they use regional samples rather than covering the country as a whole and/or in that they do not explore treatment. Thus crucial questions, such as how limitations on usual activities due to chronic pain disproportionally affect social groups in the country and which types of treatment those with chronic back pain are using, remain. Answers to these questions could shape effective public health interventions and service delivery [29].

Such interventions should be based on the understanding that a dynamic interaction of biological, psychological, and social risk and protective factors influence chronic pain [30]. The biopsychosocial model is a recognized heuristic approach to understanding the impact of chronic pain, acknowledging that a complex interaction of factors influences individuals’ reports and experiences of pain [30]. Miaskowski and colleagues’ [31] refinement of the model to examine chronic pain among older adults will be used here. It includes a number of key factors, such as biological factors (biological and chronological age, sex, comorbidity, health behaviors, fatigue and sleep disturbance), psychological (depression, anxiety, stress, substance use and abuse, pain-specific psychological factors), and social factors (race/ethnicity, culture, socioeconomic status, ageism and elder abuse, social support, and social isolation), which affect the risk of chronic back pain in older adults [10, 30, 31].

People with chronic back pain often experience difficulties in functional capacity and performing activities of daily living [32–34]. As multiple studies have shown, people who experienced chronic pain were less likely or no longer able to participate in various activities such as walking, exercising, lifting, recreation activities, conducting heavy household chores, maintaining independent lifestyles, and attending social activities [32, 34, 35]. People living with back pain report that it severely affects their emotional, sleeping, and energy functions [35].

A biopsychosocial framework is well suited to identifying non-drug as well as non-surgical treatments such as self-management, physical therapies (exercise, massage, spinal manipulation), psychological therapies (cognitive behavioral therapy), and complementary therapies (yoga, tai-chi, acupuncture) [36, 37].

Brazil’s Pesquisa Nacional de Saúde (PNS, National Health Survey) has been investigating the prevalence of chronic back pain since 2013. The current study uses the second wave of data, the PNS-2019, and the adapted biopsychosocial model to examine the following questions with respect to adults 20 and older. 1) Is chronic back pain associated with biological, psychological, and social factors? 2) How prevalent is chronic back pain? 3) Do biological, psychological, and social factors influence limitations on usual activities associated with chronic back pain? 4) How do such factors relate to the use of pharmacological and non-pharmacological treatment services among adults in Brazil?

Based on previous studies [5, 12] and the adapted biopsychosocial model [31], we hypothesize that biological factors, such as older age, being female, having more chronic conditions, and a history of smoking will be associated with a higher prevalence of chronic back pain as well as more limitations on usual activities. Psychological factors, such as having more depressive symptoms, and lower socioeconomic conditions, will be associated with higher odds of having chronic back pain and limitations on usual activities. Having higher socioeconomic status will be associated with higher odds of reporting treatment.

## Methods

### Data

This study used microdata from PNS-2019, a national health survey conducted by the Instituto Brasileiro de Geografia e Estatistica (IBGE, Brazilian Institute of Geography and Statistics). IBGE is a federal agency responsible for the official collection of statistical information in Brazil–essentially, the Brazilian census bureau [38]. PNS-2019 is a household-based national representative survey of residents living in private households [39]. PNS-2019 provides detailed information on the health conditions and determinants and the health care needs of the Brazilian population, which are used in the Brazilian health surveillance program [38, 39].

PNS employs a three-stage conglomerate plan with primary sampling units (PSU) stratification [38]. In the first stage, PSUs were selected using a proportional probability for the main sample from census tracts (or sets of tracts). In the second stage, households were randomly chosen from the tracts selected using the most recent National Register of Addresses for Statistical Purposes. In the final stage, one selected adult (15 years or older) was randomly chosen to respond to the complete questionnaire [38].

The PNS questionnaire has three parts [39]. The first part focuses on household characteristics. The second part focuses on socioeconomic conditions of all household members, such as income, level of education, health insurance coverage, health service use, and health conditions of older adults aged 60 and over and children under the age of 2. An adult member of the household provides this information regarding members of the household [39]. By simple random sampling from the list of adults in the household aged 15 or over, a respondent is selected for the third part [39]. This person provides information about their health conditions and lifestyle, among other topics [38]. The data used here was gathered for part three. The Fundação Instituto Oswaldo Cruz has detailed information about the PNS, including questionnaires, survey design, and datasets (www.pns.fiocruz.br). The microdata can be found on the IBGE website (https://www.ibge.gov.br/estatisticas/downloads-estatisticas.html?caminho=PNS/2019/Microdados/Dados).

### Ethics statement

The National Research Ethics Committee (Conselho Nacional de Ética em Pesquisa—CONEP) of the National Health Council (Conselho Nacional de Saúde–CNS) (No. 3.529.376) approved the PNS-2019 methodology. Participation was voluntary, and participants orally consented to participate [38]. The questionnaire could be answered in whole or in part. The PNS-2019 dataset is publicly available on the IBGE website without information that could identify individual respondents.

### Participants

**Fig 1:**
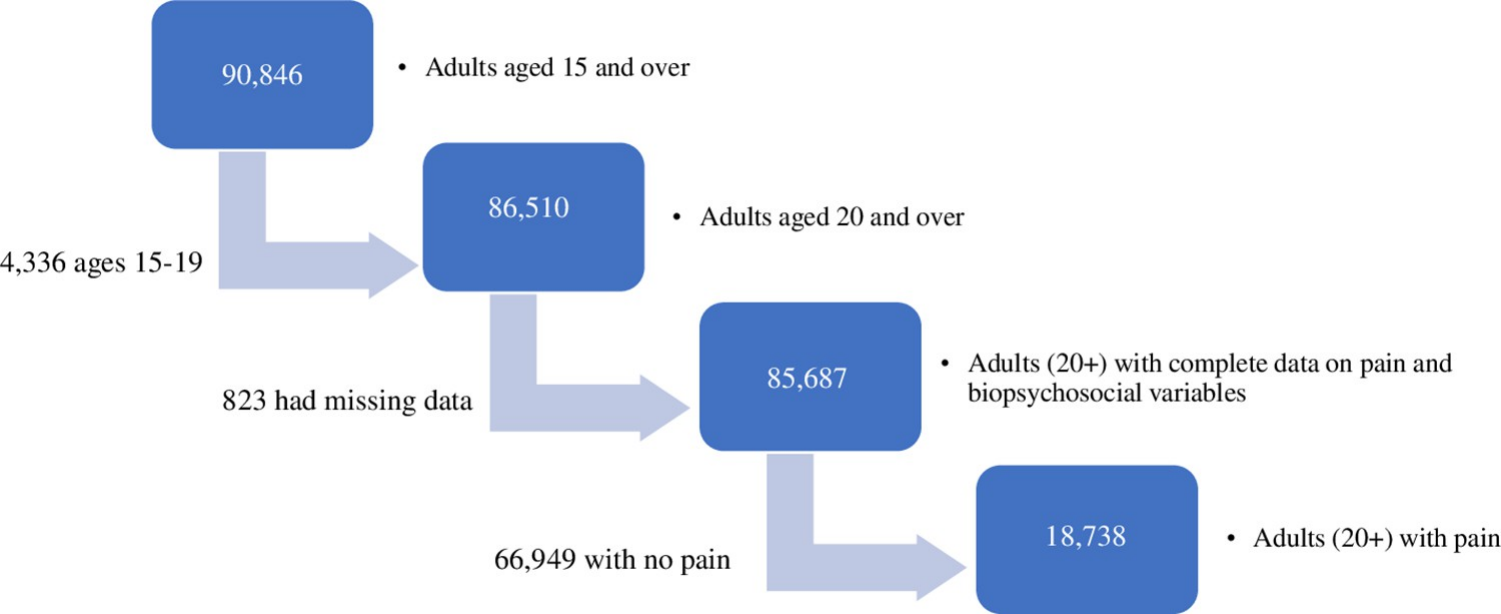
Participants flowchart in the PNS-2019.

The PNS-2019 collected data on 90,846 selected residents aged 15 and over. Eliminating respondents aged 15–19 yielded a sample of 86,510. Among these, 823 (0.95%) had missing data on selected variables. Thus, the final sample consists of 85,687 who provided complete data on selected variables. Among them, 18,738 reported having chronic back pain, and 66,949 reported that they did not. Among the 18,738, 17% reported having limitations on usual activities due to pain and 68% reported receiving at least one form of treatment (Fig 1).

### Measures

#### Pain

Pain was measured with a single question. Respondents were asked “Do you have any chronic back problems, such as chronic back or neck pain, low back pain, sciatica, vertebrae or disc problems?” (“O(a) Sr(a) tem algum problema crônico de coluna, como dor crônica nas costas ou no pescoço, lombalgia, dor ciática, problemas nas vértebras ou disco?”). Response categories were yes or no.

#### Pain treatment

Participants who had indicated they have chronic back pain reported on what they were currently doing for back pain treatment–“What are you currently doing because of your back problem?” (“O que o(a) Sr(a) faz atualmente por causa do problema na coluna?”). Categories of activities included: a. exercise regularly, b. physical therapy, c. using medications or injections, d. acupuncture, medicinal plants and herbal medicine, homeopathy, meditation, yoga, tai chi, or some other integrative and complementary practice, and e. regular follow-up with a health professional.

#### Limitations on usual activities

Finally, participants who had indicated they have chronic back pain responded to the question, “In general, to what degree does the back/spine problem limit your usual activities (such as working, doing housework, etc.)?” (“Em geral, em que grau o problema de coluna limita as suas atividades habituais (tais como trabalhar, realizar afazeres domésticos etc.?”). The response categories were: it does not limit, a little, moderately, intensively, and very intensively. Those who answered very intensively and intensively were classified as having “intense limitations on usual activities,” and the rest were categorized as not having intense limitations.

#### Biological variables

Biological variables include age, sex, chronic conditions, BMI and health behaviors (smoking, and drinking). Age was included as a categorical variable in years (20–34, 35–49, 50–64 and 65 and over), and a dichotomous variable for female (male as the reference group). PNS personnel asked the respondents, “Has a doctor given you a diagnosis of (health condition)?” Those who responded affirmatively were considered as having the chronic condition, and those who responded negatively as not having it. Chronic conditions included arthritis, hypertension, diabetes, high cholesterol, heart disease, asthma, chronic lung diseases (chronic bronchitis, emphysema or chronic obstructive pulmonary disease), cancer, and kidney failure. Individuals were categorized as having none, one, two, or three or more conditions. Participants indicated whether they currently smoked. Categories include 1. yes daily, 2. yes, less than daily, and 3. do not smoke currently. Three categories were created for alcohol consumption based on how often and how many drinks were consumed: 1. never drink, 2. drink sometimes (less than once a month), and 3. drink often. This last category includes those who reported drinking once a month or more and drank more than five drinks on one occasion in the last month. Participants self-reported their weight and height. BMI was calculated as weight (kg)/height (m^2^) and BMI categories were created: underweight, <18.5 kg/m^2^; normal, 18.5 to 24.9 kg/m^2^; overweight, 25.0 to 29.9 kg/m^2^, and obese: ≥30.0 kg/m^2^ [40].

#### Psychological variables

For the evaluation of depression, we used the Brazilian version of the Patient Health Questionnaire (PHQ-9) [41]. For each item, possible answers and respective scores are: (0) “not at all,” (1) “less than half of the days,” (2) “more than half of the days,” and (3) “almost every day.” Total PHQ-9 scores were obtained by summing across all questions. Depression was defined by a PHQ-9 score of 10 or higher [41].

#### Social variables

Social variables included education, income, private insurance, race, marital status, urban vs. rural residency, and region of residency. Data on two derived variables created by IBGE were included in the analysis education (level of instruction) and per-capita household income. Individuals were recategorized into four categories of education: 1. no education or primary incomplete, 2. primary complete but not high school, 3. complete high school (including some college), and 4. College degree or more. To calculate per-capita household income, the monthly gross income of all household members was included based on all sources of income. Participants were instructed to report income from paid work, retirement or pension, rent or lease, unemployment insurance, alimony, government programs, savings accounts or interest from financial investments, and other sources. The income of people whose status in the household was a pensioner, domestic worker, or relative of the domestic worker was excluded from the calculation of gross monthly household income. These groups were also excluded from the number of household members used as the denominator to study per-capita income. Last, per-capita household income brackets (in minimum wages) were obtained by dividing the average income of the household members by the Brazilian monthly minimum wage, which was $998 Brazilian reais (approximately $253.30 US dollars). Per-capita household was then categorized by its relationship to minimum wage. The categories were 1. “Less than 1/4,” 2. “1/4 to 1/2,” 3. “1/2 to 1,” 4. “1 to 2,” and 5. “2 or more.” A dichotomous variable measured the presence of private health insurance (having private insurance vs. not privately insured). Race was based on self-report. Participants were provided the following categories: Branca (White), Preta (Black), Parda (Mixed or Brown), Amarela (Asian descent), and Indigena (Indigenous). We combine those who selected Amarela and Indigena due to the small size of these groups. People who might consider themselves mixed-race due to having both Black and White parentage or ancestry in the United States typically consider themselves Parda in Brazil. However, factors such as social class and geographic location influence racial classifications in Brazil as well [42]. Marital status has three categories: 1. married, 2. divorced, separated, or widowed, and 3. single. Finally, we included two variables that capture broader socioeconomic context–a dichotomous variable for urban (urban vs. rural residence) and regions (North, Northeast, Midwest, Southeast, South).

### Statistical analysis

Descriptive statistics, medians, and proportions appear in Table 1. Bivariate analyses were used to examine whether those with or without pain differ on biological, psychological, and social domains. Chi-square tests were used for categorical variables and non-linear least squares estimation for age. Table 2 presents age-adjusted prevalence rates. Age-adjusted prevalence rates were obtained using direct standardization with the standard weights from the age distribution of the population aged 20 and over in 2019. Commands stdize and stdweight were used to generate the estimates. Table 3 shows the results of the multivariate logistic regressions that examine the likelihood of pain according to the domains. We provide results for three hierarchical models. Model 1 includes the biological variables, model 2 adds the psychological variable, and model 3 adds the social variables. Next, we examined how these variables influence the limitations on usual activities among individuals with chronic back pain. Results are presented in Table 4 and include all biopsychosocial variables. Finally, we examined whether biopsychosocial variables influence the odds of reporting getting treatment for chronic back pain.

**Table 1:** Descriptive statistics of the sample according to back pain status.

Variables	No pain (n = 66,949)	Pain (n = 18,738)	Total (n = 85,687)	p-value
***Biological***				
Age (%)				<0.0001
20–34	27.3	12.2	23.8	
35–49	31.2	27.7	30.4	
50–64	24.3	34.4	26.6	
65+	17.2	25.7	19.2	
Sex (%)				
Female	51.0	60.3	53.2	<0.0001
Male	49.0	39.7	46.8	
Chronic conditions (%)				<0.0001
None	58.7	35.5	53.2	
1	24.8	27.9	25.5	
2	10.8	18.6	12.7	
3 or more	5.8	18.1	8.6	
Current smoker (%)				<0.0001
No	87.0	85.3	86.6	
Yes	13.0	14.7	13.4	
Alcohol consumption (%)				<0.0001
Never drink	56.8	61.4	57.9	
Drink sometimes	27.9	26.5	27.5	
Drink often	15.4	12.1	14.6	
Body mass index categories (%)				<0.0001
Underweight	1.8	1.8	1.8	
Normal weight	39.4	34.7	38.3	
Overweight	38.0	37.8	37.9	
Obese	20.8	25.8	22.0	
***Psychological***				
Depressive symptoms (%)				<0.0001
No	91.9	80.0	89.1	
Yes	8.1	20.0	10.9	
***Social***				
Education (%)				<0.0001
No education or primary incomplete	34.86	49.56	38.29	
Primary complete or high-school incomplete	13.82	11.04	13.17	
High-school complete	33.42	25.19	31.5	
College degree or more	17.9	14.2	17.03	
Per-capita household income (%)				<0.001
Less than 1/4	7.5	7.7	7.6	
1/4 to 1/2	12.6	12.2	12.5	
1/2 to 1	27.4	29.9	28.0	
1 to 2	28.5	27.8	28.3	
2 or more	24.1	22.3	23.7	
Private health insurance (%)				0.933
No	72.1	72.1	72.1	
Yes	27.9	27.9	27.9	
Race (%)				0.585
Branca	44.3	45.3	44.6	
Preta	11.5	11.3	11.5	
Parda	42.7	42.01	42.5	
Other	1.4	1.4	1.5	
Marital status (%)				<0.0001
Married	42.7	43.24	42.8	
Divorced/separated/widowed	17.6	25.11	19.4	
Single	39.7	31.65	37.8	
Urban/rural (%)				0.0008
Urban	86.5	84.9	86.2	
Rural	13.5	15.1	13.8	
Regions (%)				<0.0001
North	7.3	6.7	7.1	
Northeast	25.6	26.8	25.9	
Midwest	8.0	6.4	7.6	
Southeast	43.4	45.2	43.8	
South	15.7	14.9	15.6	

**Table 2:** Age-adjusted prevalence rates of chronic back pain.

Variables	Prevalence	95% CI	p-value
Total	23.4		
***Biological***			
Age (%)			<0.0001
20–34	12.3	(11.5–13.1)	
35–49	20.2	(19.1–21.3)	
50–64	30.3	(29.2–31.4)	
65+	31.2	(29.9–32.5)	
Sex			<0.0001
Females	24.9	(24.1–25.6)	
Males	19.0	(18.4–19.7)	
Chronic conditions			<0.0001
None	17.4	(16.8–18.1)	
1	24.6	(23.3–25.9)	
2	30.5	(28.4–32.6)	
3 or more	44.9	(39.6–50.2)	
Current smoker			0.0053
No	21.9	(21.3–22.4)	
Yes	23.7	(22.4–24.9)	
Alcohol consumption			0.0684
Never drink	22.3	(21.6–23.0)	
Drink sometimes	22.0	(21.1–22.9)	
Drink often	20.4	(18.9–21.9)	
Body mass index categories			<0.0001
Underweight	21.8	(18.1–25.5)	
Normal weight	20.7	(19.9–21.4)	
Overweight	21.7	(21.0–22.5)	
Obese	25.5	(24.4–26.7)	
***Psychological***			
Depressive symptoms			<0.0001
No	19.9	(19.4–20.4)	
Yes	40.6	(39.0–42.2)	
***Social***			
Education			<0.0001
No education or primary incomplete	25.2	(24.3–26.2)	
Primary complete or high-school incomplete	20.0	(18.7–21.2)	
High-school complete	20.2	(19.2–21.2)	
College degree or more	19.3	(18.1–20.5)	
Per-capita household income			<0.0001
Less than 1/4	24.9	(22.8–27.1)	
1/4 to 1/2	24.2	(22.8–25.5)	
1/2 to 1	23.6	(22.7–24.6)	
1 to 2	21.0	(20.1–21.9)	
2 or more	19.7	(18.6–20.8)	
Private health insurance			0.5941
No	22.3	(21.7–22.9)	
Yes	22.0	(21.0–23.0)	
Race			0.4563
Branca	21.8	(20.9–22.7)	
Preta	22.1	(20.9–23.4)	
Parda	22.6	(21.9–23.3)	
Other	21.5	(17.6–25.3)	
Marital status			0.0520
Married	21.6	(20.8–22.4)	
Divorced/separated/widowed	23.8	(22.2–25.3)	
Single	22.0	(21.1–22.9)	
Urban/rural			0.0006
Urban	21.8	(21.2–22.4)	
Rural	24.3	(23.0–25.6)	
Regions			<0.0001
North	22.3	(21.0–23.6)	
Northeast	23.4	(22.5–24.4)	
Midwest	19.0	(17.8–20.3)	
Southeast	22.4	(21.4–23.4)	
South	20.8	(19.7–21.9)	

**Table 3:** Adjusted odds-ratios and 95% confidence intervals on the associations between reporting chronic back pain and biopsychosocial variables.

	Model 1		Model 2		Model 3	
Variables	OR	95% CI	OR	95% CI	OR	95% CI
***Biological***						
Age (ref = 20–34)						
35–49	1.73***	(1.57–1.90)	1.75***	(1.59–1.93)	1.67***	(1.51–1.84)
50–64	2.17***	(1.99–2.38)	2.28***	(2.09–2.49)	2.09***	(1.90–2.30)
65+	1.93***	(1.75–2.14)	2.11***	(1.91–2.33)	1.80***	(1.61–2.02)
Sex (ref = female)						
Male	0.76***	(0.72–0.81)	0.82***	(0.77–0.87)	0.80***	(0.75–0.85)
Chronic conditions (ref = none)						
1	1.57***	(1.45–1.69)	1.50***	(1.38–1.62)	1.49***	(1.38–1.62)
2	2.22***	(2.02–2.45)	2.07***	(1.87–2.28)	2.06***	(1.87–2.27)
3 or more	3.86***	(3.48–4.29)	3.30***	(2.97–3.66)	3.26***	(2.94–3.62)
Current smoker (ref = No)						
Yes	1.25***	(1.16–1.35)	1.18***	(1.10–1.27)	1.14***	(1.06–1.23)
Alcohol consumption (ref = never drink)						
Drink sometimes	1.06	(0.99–1.14)	1.09**	(1.02–1.17)	1.17***	(1.09–1.25)
Drink often	1.04	(0.95–1.14)	1.05	(0.96–1.15)	1.14**	(1.04–1.25)
Body mass index categories (ref = normal weight)						
Underweight	1.02	(0.82–1.27)	0.98	(0.79–1.22)	0.94	(0.76–1.17)
Overweight	1.02	(0.96–1.08)	1.02	(0.96–1.09)	1.04	(0.98–1.11)
Obese	1.12**	(1.03–1.21)	1.11**	(1.03–1.20)	1.12**	(1.04–1.22)
***Psychological***						
Depressive symptoms (ref = No)						
Yes			2.31***	(2.14–2.49)	2.31***	(2.14–2.49)
***Social***						
Education (ref = No education or primary incomplete)						
Primary complete or high-school incomplete					0.71***	(0.65–0.78)
High-school complete					0.74***	(0.69–0.80)
College degree or more					0.71***	(0.63–0.79)
Per-capita household income (ref = Less than 1/4)						
1/4 to 1/2					0.98	(0.87–1.09)
1/2 to 1					0.97	(0.87–1.08)
1 to 2					0.89*	(0.80–0.99)
2 or more					0.85**	(0.75–0.96)
Private Health Insurance (ref = No)						
Yes					1.17***	(1.08–1.26)
Race (Ref = Branca)						
Preta					0.93	(0.84–1.02)
Parda					0.96	(0.90–1.03)
Other					0.98	(0.76–1.25)
Marital status (Ref = Married)						
Divorced/Separated/Widowed					1.02	(0.95–1.10)
Single					0.96	(0.90–1.03)
Urban/rural (ref = Rural)						
Urban					0.86***	(0.79–0.94)
Regions (ref = North)						
Northeast					0.97	(0.88–1.08)
Midwest					0.76***	(0.68–0.85)
Southeast					0.94	(0.85–1.04)
South					0.85**	(0.76–0.95)

* p<0.05

** p<0.01

*** p<0.001

**Table 4:** Adjusted odds-ratios and 95% confidence intervals of having intense limitations on usual activities due to chronic back pain and biopsychosocial variables.

Variables	OR	95% CI
***Biological***		
Age (ref = 20–34)		
35–49	1.79 ***	(1.32–2.42)
50–64	1.98***	(1.49–2.63)
65+	1.70***	(1.24–2.32)
Sex (ref = female)		
Male	1.14	(0.98–1.33)
Chronic conditions (ref = none)		
1	1.45***	(1.19–1.76)
2	1.6***	(1.32–1.95)
3 or more	2.35***	(1.89–2.93)
Current smoker (ref = No)		
Yes	1.19	(0.98–1.44)
Alcohol consumption (ref = never drink)		
Drink sometimes	0.68***	(0.58–0.80)
Drink often	0.65*	(0.45–0.94)
Body mass index categories (ref = normal weight)		
Underweight	0.84	(0.54–1.31)
Overweight	1.04	(0.89–1.21)
Obese	1.33**	(1.11–1.60)
***Psychological***		
Depressive symptoms (ref = No)		
Yes	3.06***	(2.64–3.54)
***Social***		
Education (ref = No education or primary incomplete)		
Primary complete or high-school incomplete	0.90	(0.72–1.12)
High-school complete	0.76**	(0.63–0.93)
College or more	0.66**	(0.49–0.89)
Per-capita household income (ref = Less than 1/4)		
1/4 to 1/2	0.69**	(0.53–0.90)
1/2 to 1	0.65***	(0.51–0.83)
1 to 2	0.63***	(0.47–0.83)
2 or more	0.49***	(0.35–0.68)
Private health insurance (ref = No)		
Yes	0.83	(0.66–1.05)
Race (Ref = Branca)		
Preta	1.22	(0.98–1.51)
Parda	1.10	(0.96–1.26)
Other	1.18	(0.63–2.20)
Marital status (Ref = Married)		
Divorced/Separated/Widowed	0.96	(0.82–1.14)
Single	0.81*	(0.67–0.97)
Urban/rural (ref = Rural)		
Urban	0.94	(0.81–1.08)
Regions (ref = North)		
Northeast	0.90	(0.75–1.09)
Midwest	1.17	(0.91–1.49)
Southeast	1.01	(0.82–1.26)
South	1.11	(0.87–1.41)

* p<0.05

** p<0.01

*** p<0.001

Given the complex sample design of the PNS-2019, the appropriate weights derived from the complex sample design were taken into account. All analyses were performed with the survey data module (svy) of STATA package [39]. All statistical analyses were done using STATA SE 16.1.

### Inclusivity in global research

Additional information regarding the ethical, cultural, and scientific considerations specific to inclusivity in global research is included in the (S1 File).

## Results

### Bivariate associations between biopsychosocial factors and chronic back pain

Table 1 shows the descriptive statistics of biological, psychosocial, and social variables for the total sample by pain status. Most respondents (54.2%) were younger than 50 years. Women comprised more than half of the sample (53.2%). Most of the sample (53.2%) did not report having a health condition. The percentage of current smokers was 13.4%, and 14.6% reported drinking often. Most of the sample was overweight (37.9%) or obese (22%). About 1 in 10 reported having depressive symptoms.

Regarding social variables, 38.3% reported having no education or less than complete primary, and almost half lived with less than one minimum wage job per capita a month. About 28% had private health insurance. Most of the sample report being White (44.6%) or Pardo (42.5%), and 42.8% were married. The vast majority of the sample lived in urban areas, and 43.8% lived in the Southeast region. Compared to those with no pain, individuals with pain were older and more likely to be women. They were also more likely to have lower educational levels and per capita household income. A higher proportion of those with pain reported being divorced, separated, or widowed. In terms of health behaviors, participants with pain were more likely to be obese, smoke currently, and never consume alcohol. Individuals with back pain were more likely to have multiple health conditions than those with no back pain.

### Age-adjusted estimates of the prevalence of chronic back pain across groups

The prevalence of back pain in the sample was 23.4% (95% CI 22.8–24.0). Being female, with low education, overweight or obese, currently smoking, having depressive symptoms and more chronic conditions, and living in rural areas correlate positively with chronic back pain. Prevalence rates were lower among residents in the Midwest region (Table 2).

### Adjusted associations between biopsychosocial variables and chronic back pain

Table 3 presents the results of the logistic regression models. In all models, compared to those ages 20–24, older age groups had higher odds of having chronic back pain. Men were less likely than women to report having chronic back pain. Having more chronic conditions was also associated with higher odds of having chronic back pain. Adults who were current smokers had higher odds of having pain. For alcohol consumption, coefficients turned significant after adding psychological and social domains. In Model 3, those who drank sometimes or often were more likely to report having pain than those who never drank. Obesity was associated with higher odds of having chronic back pain. Having depressive symptoms was associated with higher odds of reporting having chronic back pain. Compared to those with no education or primary school incomplete, those with higher levels of education had lower odds of reporting having chronic back pain. Adults with per capita household income above one minimum wage job were less likely than those receiving less than ¼ to report having chronic back pain. Those with private health insurance were more likely to report chronic back pain. There were no statistically significant racial or marital status differences. Living in rural areas is associated with higher odds of having chronic back pain than living in urban areas. Compared to those in the North region, residents in the South and Midwest regions had lower odds of having chronic back pain.

### Biopsychosocial factors and limitations on usual activities

Next, we examine whether biological, psychological, and social variables influence the reporting of severe limitations on usual activities resulting from back pain. Among those who reported having back pain, 17% said that it intensively limited their usual activities. Higher odds of having limitations resulting from having back pain were found among older adults with more chronic conditions or obesity. Adults who drank sometimes or often had lower odds of reporting having limitations on usual activities. Having depressive symptoms was positively associated with having limitations on usual activities due to pain. Higher education and income were associated with lower odds of reporting limitations. According to private insurance, urban living, or regions, there were no differences. Single adults were less likely to report having limitations on usual activities (Table 4).

### Associations between biopsychosocial variables and the use of treatment services

Next, we examine the treatment of back pain. Among those reporting back pain, 68% received at least one form of treatment. As percentages of those receiving one or more treatment, 28% exercised regularly, 13% went to physical therapy, 45% took medications or injections, 7% got alternative treatments, such as acupuncture, and 27% visited a health professional regularly. Table 5 shows the results of the logistic regression for the overall treatment. Results indicated that compared to those 20–34, adults ages 35–64 were more likely to receive some treatment for their back pain. Being male and currently smoking were associated with lower odds of treatment. The odds of receiving treatment increased with more chronic conditions. In general, higher socioeconomic conditions, such as college education and having private insurance, were associated with higher odds of receiving treatment. Adults living in urban areas were more likely to get treatment. Living in the Southeast region was associated with lower odds of any treatment.

**Table 5:** Adjusted odds-ratios and 95% confidence intervals on the associations between getting treatment for pain and biopsychosocial variables.

Variables	OR	95% CI
***Biological***		
Age (ref = 20–34)		
35–49	1.25*	(1.04–1.49)
50–64	1.41***	(1.18–1.69)
65+	1.22	(0.98–1.50)
Sex (ref = female)		
Male	0.73***	(0.65–0.82)
Chronic conditions (ref = none)		
1	1.20*	(1.04–1.39)
2	1.25**	(1.06–1.46)
3 or more	1.47***	(1.23–1.75)
Current smoker (ref = No)		
Yes	0.83*	(0.71–0.97)
Alcohol consumption (ref = never drink)		
Drink sometimes	0.95	(0.83–1.08)
Drink often	1.14	(0.96–1.36)
Body mass index categories (ref = normal weight)		
Underweight	0.74	(0.53–1.03)
Overweight	1.03	(0.92–1.16)
Obese	0.98	(0.84–1.15)
***Psychological***		
Depressive symptoms (ref = No)		
Yes	1.08	(0.94–1.24)
***Social***		
Education (ref = No education or primary incomplete)		
Primary complete or high school incomplete	1.03	(0.87–1.22)
High school complete	1.12	(0.97–1.30)
College or more	1.40**	(1.12–1.74)
Per-capita household income (ref = Less than 1/4)		
1/4 to 1/2	1.13	(0.93–1.38)
1/2 to 1	1.2	(0.99–1.44)
1 to 2	1.24*	(1.02–1.51)
2 or more	1.25	(0.98–1.59)
Private health insurance (ref = No)		
Yes	1.34***	(1.14–1.57)
Race (Ref = Branca)		
Preta	0.85	(0.71–1.02)
Parda	0.96	(0.85–1.08)
Other	0.98	(0.57–1.68)
Marital status (Ref = Married)		
Divorced/Separated/Widowed	1.07	(0.92–1.23)
Single	0.97	(0.85–1.10)
Urban/rural (ref = Rural)		
Urban	1.16*	(1.02–1.31)
Regions (ref = North)		
Northeast	0.9	(0.79–1.03)
Midwest	1.05	(0.87–1.26)
Southeast	0.82*	(0.70–0.96)
South	0.95	(0.80–1.12)

* p<0.05

** p<0.01

*** p<0.001

S1 Table provides the results for each type of treatment. In terms of biological conditions, results show that, in general, except for exercise and alternative methods, older age is associated with receiving physical therapy, medications/injections, and regularly visiting doctors. Men are less likely than women to receive all types of treatments. On the other hand, morbidity and multimorbidity are associated with higher odds of treatment. The only statistical difference between current smokers and non-smokers is exercise, as smokers are less likely to exercise. Results are less clear for alcohol consumption. Those who drink sometimes are more likely to exercise and use alternative methods but less likely to use medications/injections. In contrast, those who drink often are more likely to exercise but less likely to have regular visits. Results for BMI indicate that underweight adults are less likely to exercise and have regular visits. Obese adults are more likely to receive physical therapy, whereas overweight adults are less likely to use alternative methods than adults with normal BMI.

In terms of psychological conditions, adults with depressive symptoms were less likely to exercise but more likely to receive physical therapy, use medications/injections, and have regular health visits. Regarding social conditions, higher education, particularly having college degree or more, was positively associated with exercise, physical therapy, and alternative methods but negatively associated with medications and injections. There were clear gradients in income, with those with higher income engaging in exercise, physical therapy, and regular health visits. Having private insurance and living in urban areas increased the odds of receiving physical therapy and regular visits. Those in urban areas were also more likely to exercise. There were few regional differences–adults in the Midwest were more likely to have regular health visits, but those in the Southeast were less likely to use medications and injections. Those in the Northeast were less likely to receive alternative treatments.

## Discussion

This study uses the National Survey of Health data to derive a comprehensive overview of how biopsychosocial factors are associated with the prevalence, limitations, and treatment of chronic back pain in Brazil. We find important differences across biological, psychological, and social variables in the prevalence of chronic back pain, limitations on usual activities, and treatment utilization among adults in Brazil.

Chronic back pain has a significant impact on the adult population in Brazil. While the overall prevalence rate was 18.5% in 2013 [5], six years later, it had increased to 23.4%. Back pain has major social costs. Direct health costs associated with spinal disorders, including inpatient and outpatient care, amounted to US$71 million in 2016 [43]. Most (58%) of the direct health costs incurred in Brazil to treat spinal disorders can be attributed to inpatient care, such as hospitalizations, medications, and diagnostic tests [43].

The majority of those with chronic back pain do not become severely limited in their usual activities of daily living. However, 17% of those who reported having back pain had severe limitations. These limitations can result in disability and economic productivity loss. In The number of working days lost to disability due to chronic back pain was more than 12 million in 2007 [44]. Back pain is the main cause of disability among people who retire due to injury and thus costs the nation dearly in lost productivity and social security pensions [44].

Current guidelines have proposed that back pain treatment should involve patient education, supervised exercise, and therapy [45, 46]. However, The most common reported treatment among those with back pain in Brazil was medications (45%). Only 28% of adults with back pain reported exercising and even fewer, 13%, engaged in physical therapy. Nonetheless, costs related to physical therapy represented the largest part of outpatient expenses related to the treatment of spine disorders in 2016 in Brazil, representing 20% of the total direct costs [43].

Current guidelines for the prescription of medication depend on the duration of the pain and most recommend the use of nonsteroidal anti-inflammatory drugs [46]. Injections and opioids should be taken with caution [45, 46]. One of the limitations of the PNS-2019 is that it does not distinguish among types of medication.

Questions also remain about the 32% of people who experience chronic pain but were not receiving any treatment. It is possible that these respondents had moderate pain that they could manage on their own or had problems with their treatments and discontinued them. More information is needed on why they are not receiving treatment [32]. Given the disparities in treatment, additional strategies to reach those with lower treatment levels, such as men and those with lower education, are needed.

### Biological factors

The prevalence of chronic back pain increased with age. Age-related physical changes to muscles, bones, and joints become more common at older ages. In addition to these physical changes, evidence suggests that the alterations in pain perception, central pain mechanisms, and neuroplastic changes contribute to pain responses [47–49]. Compared to those 20–34, adults ages 35 and older were more likely to report limitations on usual activities, but treatment peaked in the group ages 50 to 64. For the oldest group (65+), it is possible that they may accept pain as a normal part of the aging process and hold low expectations of treatments [48], particularly exercise.

Women were more likely to report chronic back pain than men. The observed association in our study was consistent with other studies undertaken in Brazil [5], Japan [8], and other European countries [7, 50, 51], demonstrating greater pain prevalence among women than men [10, 52, 53]. A significant number of studies have indicated that men and women differ in their responses to pain, with women tending to show increased pain sensitivity and risk for clinical pain [11, 52, 54]. These responses may be due to biological conditions associated with pregnancy and childbearing, the physical stress of child-rearing, perimenopausal abdominal weight gain [55], and menstrual cycle fluctuations [56]. In addition, psychological and social processes that differ between men and women might affect the perception, expression, and tolerance of pain, as well as coping strategies [10]. Yet, there were no statistical differences between men and women in reporting limitations on usual activities. Women were also more likely to report receiving treatment than men. Women use more outpatient care, such as healthcare consultations, physiotherapy, and acupuncture, and men have higher inpatient care [43]. It is possible that men seek assistance when the condition is more severe [43], which may lead to higher costs related to inpatient care and disability. Indeed, Brazilian men have a higher rate of receiving a disability pension due to back pain than women [44].

Regarding chronic health conditions, there is a gradient in which those with more conditions are more likely to have chronic pain, and, among those with pain, those with growing multimorbidity are more likely to have severe limitations and to be in treatment. Individuals with back pain often have comorbid conditions [57]. Pulmonary diseases [58] and diseases that affect nerve conduction, such as fibromyalgia [59], are the most common chronic conditions accompanying pain.

Our findings concerning being obese likewise align with past research in that those who are obese are more likely to report having back pain and also more likely to report having severe limitations on usual activities [5, 60]. For example, a study of Australian men demonstrated that lower back pain was associated with higher BMI, and obesity was associated with high levels of back pain and disability [18]. Obesity may have biomechanical and meta-inflammatory effects on the spine [60], and systemic metabolic processes associated with excess adipose tissue may also play a role in back pain [18]. Among those with chronic back pain, obesity did not predict treatment services, except that people with obesity were more likely to receive physical therapy. Obesity seems to prolong recovery and require more physical therapy sessions [61]. Since those who are obese have more back pain, obese people are likely undertreated.

Our study also revealed that current smokers had a higher risk of having chronic back pain, which also accords with several studies, including in Brazil [5, 13, 62]. Among those with chronic back pain, smokers did not report higher levels of limitations, which may be related to nicotine’s analgesic properties [31]. However, they were less likely to be treated, particularly to exercise. In general, adults who smoke in Brazil are less likely to exercise A meta-analysis comparing the prevalence of chronic pain among former smokers, never smokers, and current smokers showed that current smokers have the highest prevalence of low back pain and never smokers have the least [13]. Smoking impairs fibrinolysis and promotes fibrin deposition and scar formation, which may cause back pain [63]. Smoking may also increase coughing activity and thus increase intradiscal and intra-abdominal pressure, leading to a burden on the spine [64]. Smoking also differs across social groups in Brazil as elsewhere, being higher among those with lower socioeconomic status, such as those with lower education and older income [65, 66]. Smoking is also associated with older age and being a single man [66]. In our analyses, the coefficient for smoking in Table 3 decreased as social variables were added to the model, but it remained significant in the association with the prevalence of chronic pain.

A systematic review of the research suggests that alcohol consumption correlates with a higher risk of chronic back pain, but this impact is not consistent across studies [14]. Our findings thus aligned with past research showing that those who drink are more likely to have pain [31], but we also report that they are less likely to have limitations. It is possible that people use alcohol to relieve pain. The associations between alcohol consumption and pain treatment in our findings are complex. For instance, alcohol consumption was associated with exercising and alternative treatment methods, but negatively associated with regular health visits. Further examination is needed to better understand these correlations.

### Psychological factors

Among those with chronic back pain, 20% reported having depressive symptoms. This prevalence is similar to that found in a study of adults in Europe [32]. Other studies with adult populations have also found a correlation [19, 25]. Depressive symptoms, anxiety, and sleep disorders are common sequelae of back pain [57], but people experiencing depression are also more likely to develop back pain in their lifetime than those without depression [25]. Among older women in Brazil, depressive symptoms were associated with severity of pain [67]. Brazilian adults with depressive symptoms were three times as likely as those without to report limitations on usual activities. This positive association with limitations and disability is also found in other studies [67, 68].

### Social factors

Consistent with previous research findings, higher education and income correlated with less chronic back pain [19, 20, 51]. Brazilians who have high school or more education were less likely to suffer from intense limitations and more likely to utilize treatment services, particularly exercise, physical therapy, medications/injections, and having regular doctor’ visits. The income association was apparent for those with income above one minimum wage per capita, in which increases in income were associated with a lower likelihood of back pain. Similar findings were also found in PNS-2013 [5] and other countries [8, 17, 19, 69]. Research provides a range of possible explanations for this correlation. People with lower socioeconomic status are more likely to live in hazardous environments and have strenuous jobs [21, 54]. A review of studies conducted worldwide found that those in the highest groups of education and income status were more likely to be aware of the risk of chronic back pain (and, thus, presumably, how to avoid them) and also to seek and receive treatments [21]. Research in the United States suggests that jobs that require physically demanding tasks cause stresses on the spine, but people with pain cannot afford to quit their jobs [70]. In Brazil, back pain is the leading cause of disability among social security pension and retirement [44].

Rural residents showed a higher risk of having chronic back pain, which may be associated with more physically demanding work [5]. This study supports previous observations of rural and urban variation, including in Brazil [5, 51, 71]. Nonetheless, there were no differences among residents in rural versus urban areas regarding reporting limitations on usual activities. This contrasts with findings that show that most pensioners, due to back pain, live in urban areas in Brazil [44]. On the other hand, residents in urban areas were more likely to report all types of treatments, except for alternative methods. Residents in urban areas tend to be more educated, which may explain their higher use of health care.

Brazil is divided into five regions politically and geographically, and populations in each region have different demographic and socioeconomic characteristics [72]. South, Southeast and Midwest are the most advanced in levels of human development. PNS-2019 shows that adults in these regions differ in health status in line with past research [27, 72]. Residents in the Midwest and South regions were less likely than those in the North, Northeast, and Southeast to report having back pain. An internet-based survey of adults in Brazil found higher prevalence of chronic pain in the South, Northeast, and Midwest [73]. Further studies are needed to better understand these regional disparities as differences may emerge due to sampling, definitions, or variables included in the analyses. Nonetheless, our study showed that the likelihood of reporting limitations was similar in each of the five regions. On the other hand, residents of the Southeast were less likely to be receiving treatment, especially medication of any kind. This contrasts with reports of greater access to health care service in the Southeast region, but may reflect that this region has one of the lowest prevalence rates of prescribed medicine during health care visits [74]. Finally, adults with back pain in the Midwest were more likely to report regular health visits.

### Limitations

The present study has some limitations. The PNS-2019 contains very few questions regarding each participants’ pain experience. In addition, the pain variable was self-reported and did not follow the most updated clinical diagnostic criteria. Similarly, there was a single question about limitations due to back pain, which can be subjective. Validated measures such as the Rolland-Morris disability questionnaire or the Oswestry disability index would be superior [75]. Moreover, the single question does not allow us to examine how pain interferes with specific usual tasks, as well as personal care [68]. Data on treatment also does not distinguish between types of medications. The data are all self-reported and thus prone to social desirability bias for variables such as drinking and smoking. Health variables can also have reporting problems as access to healthcare and diagnostic testing vary across social groups. Another limitation is the cross-sectional data design, which does not allow for causal inferences. The 823 respondents who participated in all parts of the survey but had missing data on selected variables were younger, more likely to be women, and less likely to report having back pain than those with complete data, which might introduce some bias. Finally, the PNS-2019 is a household-based survey. It does not include individuals who live in long-term care facilities for older adults, which may bias results [39].

## Conclusions

Chronic back pain affects more than one-fifth of Brazilian adults. About 17% of those who reported having back pain had severe limitations and about a third did not receive any treatment. There are significant disparities across biological, psychological and social domains in the prevalence of chronic back pain, limitations on usual activities, and treatment. This research should be used to guide interventions to prevent the development of chronic back pain and to address disparities so that all Brazilians have the opportunity to live with as little limitation from back pain as possible.

## Supporting information

S1 Table Adjusted odds-ratios and 95% confidence intervals on the associations between getting types of treatment for pain and biopsychosocial variables. * p<0.05, ** p<0.01, *** p<0.001.
(DOCX)

S1 File (DOCX)
