# Supplementary material for: A biopsychosocial examination of chronic back pain, limitations on usual activities, and treatment in Brazil, 2019
Source: PLoS One. 2022 Jun 3;17(6):e0269627. doi: 10.1371/journal.pone.0269627 (PMC9165836; doi:10.1371/journal.pone.0269627)
Supplement: S1 Table — * p<0.05, ** p<0.01, *** p<0.001. (DOCX) [file pone.0269627.s001.docx]

|  | Exercise | | Physical Therapy | | Medications/injections | | Alternative methods | | Regular visits | |
| --- | --- | --- | --- | --- | --- | --- | --- | --- | --- | --- |
| Variables | OR | 95% CI | OR | 95% CI | OR | 95% CI | OR | 95% CI | OR | 95% CI |
| ***Biological*** |  |  |  |  |  |  |  |  |  |  |
| Age (ref=20-34) |  |  |  |  |  |  |  |  |  |  |
| 35-49 | 0.97 | (0.78 - 1.21) | 1.63** | (1.19 - 2.25) | 1.30** | (1.09 - 1.56) | 1.17 | (0.86 - 1.58) | 1.64*** | (1.33 - 2.04) |
| 50-64 | 0.91 | (0.73 - 1.13) | 1.83*** | (1.32 - 2.53) | 1.42*** | (1.19 - 1.68) | 1.17 | (0.80 - 1.72) | 1.89*** | (1.51 - 2.37) |
| 65+ | 0.67** | (0.51 - 0.88) | 1.52* | (1.02 - 2.27) | 1.21 | (0.99 - 1.49) | 0.89 | (0.55 - 1.44) | 1.63*** | (1.26 - 2.10) |
| Sex (ref=female) |  |  |  |  |  |  |  |  |  |  |
| Male | 0.86** | (0.77 - 0.96) | 0.81* | (0.68 - 0.97) | 0.76*** | (0.68 - 0.85) | 0.64*** | (0.51 - 0.81) | 0.77*** | (0.68 - 0.88) |
| Chronic conditions (ref=none) |  |  |  |  |  |  |  |  |  |  |
| 1 | 1.19* | (1.01 - 1.41) | 1.31** | (1.08 - 1.59) | 1.17* | (1.04 - 1.33) | 1.37* | (1.05 - 1.79) | 1.34*** | (1.15 - 1.56) |
| 2 | 1.32** | (1.09 - 1.60) | 1.74*** | (1.32 - 2.30) | 1.32*** | (1.14 - 1.54) | 1.68*** | (1.25 - 2.26) | 1.57*** | (1.30 - 1.91) |
| 3 or more | 1.27* | (1.03 - 1.56) | 1.94*** | (1.49 - 2.53) | 1.59*** | (1.34 - 1.87) | 1.26 | (0.83 - 1.90) | 1.86*** | (1.55 - 2.21) |
| Current smoker (ref=No) |  |  |  |  |  |  |  |  |  |  |
| Yes | 0.56*** | (0.46 - 0.67) | 0.89 | (0.70 - 1.12) | 1.11 | (0.96 - 1.28) | 0.99 | (0.72 - 1.37) | 0.86 | (0.72 - 1.03) |
| Alcohol consumption (ref=never drink) |  |  |  |  |  |  |  |  |  |  |
| Drink sometimes | 1.18* | (1.01 - 1.38) | 0.98 | (0.81 - 1.18) | 0.83** | (0.73 - 0.94) | 1.29* | (1.05 - 1.59) | 0.96 | (0.84 - 1.10) |
| Drink often | 1.28** | (1.06 - 1.54) | 0.8 | (0.60 - 1.06) | 0.97 | (0.81 - 1.17) | 1.3 | (0.93 - 1.82) | 0.72* | (0.55 - 0.94) |
| Body mass index categories (ref=normal weight) |  |  |  |  |  |  |  |  |  |  |
| Underweight | 0.40** | (0.21 - 0.76) | 0.62 | (0.33 - 1.18) | 0.85 | (0.62 - 1.16) | 0.95 | (0.46 - 1.98) | 0.56* | (0.34 - 0.91) |
| Overweight | 1.06 | (0.93 - 1.19) | 1.07 | (0.92 - 1.26) | 1.08 | (0.97 - 1.21) | 0.74** | (0.59 - 0.93) | 1.03 | (0.91 - 1.16) |
| Obese | 0.95 | (0.76 - 1.19) | 1.24* | (1.02 - 1.50) | 1.08 | (0.94 - 1.23) | 0.81 | (0.64 - 1.02) | 1.11 | (0.95 - 1.29) |
| ***Psychological*** |  |  |  |  |  |  |  |  |  |  |
| Depressive symptoms (ref=No) |  |  |  |  |  |  |  |  |  |  |
| Yes | 0.69*** | (0.60 - 0.80) | 1.22* | (1.02 - 1.45) | 1.40*** | (1.23 - 1.60) | 1.23 | (0.93 - 1.62) | 1.22* | (1.04 - 1.43) |
| ***Social*** |  |  |  |  |  |  |  |  |  |  |
| Education (ref=No education or primary incomplete) |  |  |  |  |  |  |  |  |  |  |
| Primary complete or high-school incomplete | 1.12 | (0.89 - 1.39) | 1.11 | (0.81 - 1.53) | 0.89 | (0.75 - 1.05) | 1.14 | (0.77 - 1.68) | 0.98 | (0.82 - 1.18) |
| High-school complete | 1.41*** | (1.21 - 1.63) | 1.2 | (0.88 - 1.64) | 0.80** | (0.70 - 0.91) | 1.32 | (0.90 - 1.93) | 1.09 | (0.93 - 1.27) |
| College or more | 1.98*** | (1.56 - 2.52) | 1.48* | (1.04 - 2.09) | 0.69*** | (0.56 - 0.85) | 1.76* | (1.12 - 2.75) | 1.22 | (1.00 - 1.48) |
| Per-capita household income (ref=Less than 1/4) |  |  |  |  |  |  |  |  |  |  |
| 1/4 to 1/2 | 1.31* | (1.02 - 1.69) | 1.54* | (1.08 - 2.19) | 1.05 | (0.86 - 1.27) | 0.77 | (0.45 - 1.32) | 1.15 | (0.88 - 1.50) |
| 1/2 to 1 | 1.55*** | (1.21 - 1.99) | 1.77** | (1.25 - 2.51) | 1.11 | (0.93 - 1.33) | 0.68 | (0.39 - 1.18) | 1.38** | (1.08 - 1.76) |
| 1 to 2 | 1.74*** | (1.37 - 2.22) | 1.80*** | (1.28 - 2.53) | 1.06 | (0.87 - 1.30) | 0.91 | (0.52 - 1.58) | 1.43** | (1.12 - 1.83) |
| 2 or more | 2.14*** | (1.61 - 2.84) | 2.75*** | (1.73 - 4.37) | 0.92 | (0.73 - 1.16) | 1.59 | (0.77 - 3.32) | 1.53** | (1.16 - 2.03) |
| Private Health Insurance (ref=No) |  |  |  |  |  |  |  |  |  |  |
| Yes | 1.15 | (0.99 - 1.34) | 1.41** | (1.14 - 1.76) | 0.88 | (0.75 - 1.04) | 1.13 | (0.83 - 1.53) | 1.78*** | (1.51 - 2.10) |
| Race (Ref= Branca) |  |  |  |  |  |  |  |  |  |  |
| Preta | 0.82 | (0.66 - 1.01) | 0.99 | (0.77 - 1.28) | 0.87 | (0.74 - 1.02) | 0.75 | (0.54 - 1.05) | 0.89 | (0.72 - 1.09) |
| Parda | 0.92 | (0.81 - 1.05) | 1.03 | (0.87 - 1.21) | 1.02 | (0.92 - 1.13) | 1.04 | (0.78 - 1.38) | 0.92 | (0.78 - 1.08) |
| Other | 0.92 | (0.58 - 1.45) | 0.77 | (0.42 - 1.38) | 0.84 | (0.56 - 1.27) | 1.55 | (0.84 - 2.88) | 0.76 | (0.46 - 1.26) |
| Marital status (Ref=Married) |  |  |  |  |  |  |  |  |  |  |
| Divorced/Separated/Widowed | 1.02 | (0.87 - 1.20) | 0.97 | (0.80 - 1.17) | 1.07 | (0.94 - 1.21) | 0.92 | (0.69 - 1.23) | 1.06 | (0.92 - 1.21) |
| Single | 0.91 | (0.79 - 1.04) | 1 | (0.83 - 1.20) | 0.97 | (0.86 - 1.10) | 0.89 | (0.68 - 1.16) | 0.9 | (0.77 - 1.05) |
| Urban/rural (ref=Rural) |  |  |  |  |  |  |  |  |  |  |
| Urban | 1.73*** | (1.47 - 2.03) | 1.52*** | (1.24 - 1.88) | 0.98 | (0.87 - 1.09) | 0.86 | (0.67 - 1.11) | 1.22* | (1.04 - 1.43) |
| Regions (ref=North) |  |  |  |  |  |  |  |  |  |  |
| Northeast | 0.92 | (0.78 - 1.08) | 0.88 | (0.71 - 1.09) | 0.98 | (0.86 - 1.12) | 0.58*** | (0.44 - 0.75) | 0.93 | (0.78 - 1.10) |
| Midwest | 0.94 | (0.77 - 1.15) | 0.88 | (0.68 - 1.13) | 0.93 | (0.77 - 1.13) | 0.91 | (0.67 - 1.23) | 1.34** | (1.10 - 1.64) |
| Southeast | 0.9 | (0.74 - 1.09) | 0.94 | (0.73 - 1.21) | 0.82* | (0.71 - 0.96) | 0.78 | (0.58 - 1.05) | 1.08 | (0.91 - 1.29) |
| South | 0.82 | (0.67 - 1.00) | 0.94 | (0.73 - 1.20) | 0.87 | (0.74 - 1.02) | 0.95 | (0.71 - 1.28) | 1.1 | (0.90 - 1.34) |
